# Molecular distributions and compound-specific stable carbon isotopic compositions of lipids in wintertime aerosols from Beijing

**DOI:** 10.1038/srep27481

**Published:** 2016-06-08

**Authors:** Lujie Ren, Pingqing Fu, Yue He, Juzhi Hou, Jing Chen, Chandra Mouli Pavuluri, Yele Sun, Zifa Wang

**Affiliations:** 1 State Key Laboratory of Atmospheric Boundary Layer Physics and Atmospheric Chemistry, Institute of Atmospheric Physics, Chinese Academy of Sciences, Beijing 100029, China; 2 University of Chinese Academy of Sciences, Beijing 100049, China; 3 Collaborative Innovation Center on Forecast and Evaluation of Meteorological Disasters, Nanjing University of Information Science & Technology, Nanjing, 210044, China; 4 Institute of Tibetan Plateau Research, Chinese Academy of Sciences, Beijing 100085, China; 5 Institute of Geographic Sciences and Natural Resources Research, Chinese Academy of Sciences, Beijing 100101, China; 6 Institute of Surface-Earth System Science, Tianjin University, Tianjin 300072, China

## Abstract

Molecular distributions and stable carbon isotopic compositions (δ^13^C) of *n*-alkanes, fatty acids and *n*-alcohols were investigated in urban aerosols from Beijing, northern China to better understand the sources and long-range atmospheric transport of terrestrial organic matter during polluted and clear days in winter. *n*-Alkanes (C_19_–C_36_), fatty acids (C_8_–C_32_) and *n*-alcohols (C_16_–C_32_) detected in Beijing aerosols are characterized by the predominance of C_23_, C_16_ and C_28_, respectively. Carbon preference index (CPI) values of *n*-alkanes, the ratios of the sum of odd-numbered *n*-alkanes to the sum of even-numbered *n*-alkanes, are close to 1, indicating a heavy influence of fossil fuel combustion. Relatively higher ratios of C_(18:0+16:0)_/C_(18:n+16:1)_ (fatty acids) on clear days than polluted days indicate that long-distance transport and/or photochemical aging are more significant during clear days. *δ*^13^C values of *n*-alkanes and low molecular weight fatty acids (C_16:0_, C_18:0_) ranged from –34.1 to −24.7% and −26.9 to −24.6%, respectively, which are generally heavier on polluted days than those on clear days. Such a wide range suggests that atmospheric lipids in Beijing aerosols originate from multiple sources and encounter complicated atmospheric processes during long-range transport in North China.

Particulate organic matter is one of the important components in atmospheric aerosols. It leads to many environmental problems such as radioactive forcing, acidic precipitation, photochemical fog and health effects^1^. Among the types of atmospheric organic matter, alkyl lipids such as *n*-alkanes, fatty acids and *n*-alcohols are ubiquitous in continental and marine aerosols, which are good tracers for investigating the origin and fate of atmospheric aerosols owing to their special characteristics^2^. For example, ambient aerosol-associated *n*-alkanes mainly originate from anthropogenic sources (e.g., incomplete combustion and lubricant oils)^3^ and biogenic sources released from the epicuticular waxes of vascular plants and the suspension of pollen, fungi, bacteria and algae^4^. Furthermore, it is well-known that high molecular weight (HMW, ≥C_26_) *n*-alkanes from vascular plants exhibit high odd-to-even predominance^5^ versus those in petroleum (C_16_–C_25_)^5^,^6^,^7^.

Fatty acids are abundant in aerosol and are found to contribute 5.8–55% of the compounds identified in meat cooking^8^,^9^, automobiles^10^, biomass combustion^11^ and leaf surfaces of urban plants^12^. As the major lipid components in aerosol, fatty acids have been identified and quantified in aerosols collected from different geographical locations, including remote marine^13^,^14^, high mountain^2^, rural^15^ and urban areas^14^,^16^,^17^. Unsaturated fatty acids in aerosols are unstable and are rapidly oxidized and degraded in the atmosphere due to reaction with radicals (OH and NO_3_) and ozone^18^,^19^. Thus, they are often used as an indicator of the apparent “aging” of the acids mainly derived from anthropogenic sources, such as cooking and biomass burning. *N*-Alcohols are one of the important typical biomarkers that originate from epicuticular waxes of terrestrial higher plants. They can be emitted from the resuspension of decaying plant leafs in soils into the atmosphere, or injected as smoke particles by biomass burning^2^,^20^. Relative abundances of *n*-alcohols, *n*-alkanes and *n*-acids of high molecular weight are used to distinguish the source regions of terrestrial biomarkers^2^.

In addition to molecular distributions, stable carbon isotopic compositions (δ^13^C) of low molecular weight (LMW) and HMW *n*-alkanes or *n*-acids also provide useful information about the origins and apportionment in natural environments. Terrestrial plants and human activities, such as fossil fuel combustion, release various organic carbonaceous compounds which are the constituents of lipids in aerosol. C3, C4 and CAM (crassulaceae acid metabolic) plants have different photosynthetic pathways. δ^13^C values of *n*-alkanes and *n*-acids in terrestrial plants are sensitive to the plant type from which they originate, due to the isotopic differences (up to 20% between C3 and C4 plants)^21^,^22^. Furthermore, the isotopic compositions of these organic carbonaceous compounds are preserved in atmospheric and geological samples without suffering major modification^23^. Thus, the compound-specific stable carbon isotope analysis (CSIA) of lipids in aerosol can determine their origin and is a powerful tool for source determination^24^,^25^,^26^,^27^.

CSIA of terrestrial biomarkers has been extensively applied to investigate potential transport processes, including agricultural soil, gasoline and diesel vehicle combustion, forest fire and street dust^28^,^29^,^30^,^31^. A previous study discriminated among the *δ*^13^C values from the rural versus agricultural soils, street dust, soot from vehicles and volcanic dust of PM_2.5_ and PM_10_, and the results indicate that *δ*^13^C values in particles of different size ranging from the same origin were essentially the same^30^. Kawamura *et al.*^31^ evaluated the contributions of modern/fossil carbon and marine/terrestrial organic matter with the method of stable isotopes; the result suggested that LMW fatty acids are predominantly from algal sources whereas HMW fatty acids are mainly derived from terrestrial C_3_ higher plants. Furthermore, studies on the *δ*^13^C of lipid compounds in aerosols can reveal long-range atmospheric transport processes^31^,^31^. These studies suggest that CSIA is a good tool to assess the potential sources and processes of aerosol samples^31^,^31^,^28^,^29^,^30^,^31^.

Beijing is one of the most economically developed megacities in China. The rapid development of urbanization and industrialization, including power generation, transportation, cooking and industrial activities, has led to increased anthropogenic emissions of atmospheric aerosols^33^. Thus, heavy aerosol loadings during haze episodes have become a serious environmental issue in Beijing and its surrounding regions over the past decade. In this study, we report the organic molecular distributions of lipid compounds including *n*-alkanes, fatty acids and *n*-alcohols in Beijing aerosols collected during wintertime. Furthermore, compound-specific stable C isotope ratios of *n*-alkanes and fatty acids are investigated and discussed with meteorological factors in order to better understand the possible sources of urban aerosols in Beijing.

## Results and Discussion

### Molecular distributions of lipid compounds

**Figure 1:**
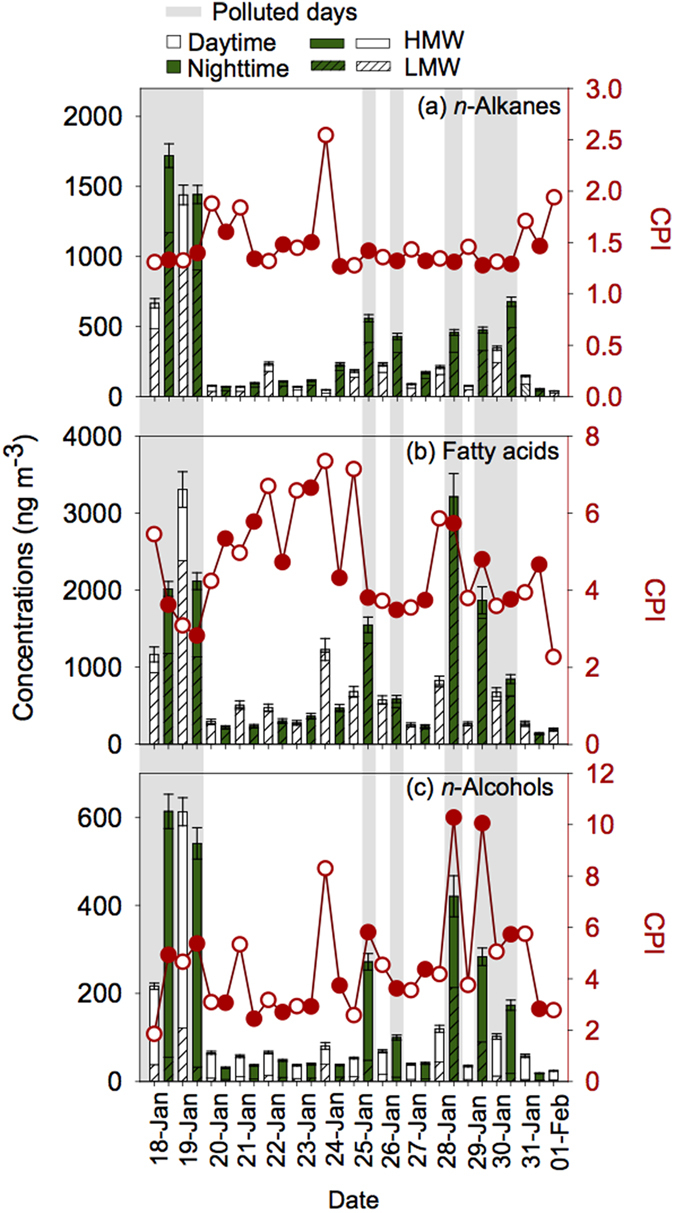
Temporal variations in the concentration of aliphatic lipids detected in Beijing aerosols.

A homologous series of *n*-alkanes (C_19_–C_36_), fatty acids (C_8_–C_32_) and *n*-alcohols (C_16_–C_32_) were detected in Beijing aerosols. The temporal variations are shown in Fig. 1 and the summary of the concentrations is presented in Table 1. Concentrations of individual compounds are provided in Table S1 of the supporting information (SI).

#### n-Alkanes

**Figure 2:**
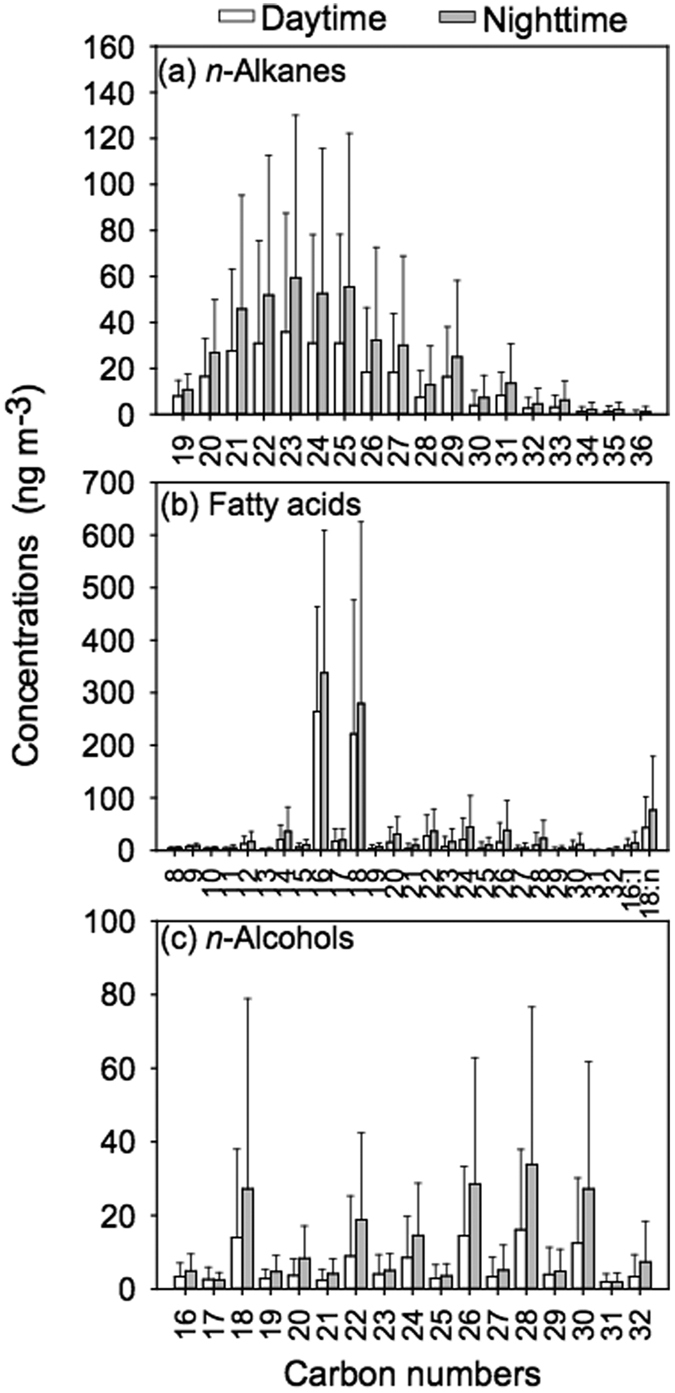
Molecular distributions and average concentrations of (**a**) *n*-alkanes, (**b**) fatty acids and (**c**) *n*-alcohols in aerosol samples from Beijing.

Concentrations of *n*-alkanes (C_19_–C_36_) range from 40.1 to 1720 ng m^−3^ (average 367 ng m^−3^) (Table 1). These concentrations are comparable to those (573 ng m^−3^) reported in wintertime samples in fourteen Chinese megacities^34^ and are higher than those (177 ng m^−3^) in summertime Mt. Tai aerosols^2^. Furthermore, the concentrations of *n*-alkanes in Beijing aerosols are 2 orders of magnitude higher than those (0.11–14.1 ng m^−3^) in marine aerosols^27^,^35^. The temporal variations of *n*-alkanes have been shown to exhibit a noticeable increase in nighttime (Fig. 1a). Concentrations of *n*-alkanes in nighttime (55.3–1720 ng m^−3^, 472 ng m^−3^) were nearly two times higher than those (40.1–1440 ng m^−3^, 262 ng m^−3^) in daytime (Table 1; Fig. 2a), although the diurnal variations are not significant (Table S2). Homologous *n*-alkanes are characterized by no odd/even predominance in the range of low molecular weight (LMW, <C_26_) *n*-alkanes and weak odd carbon number predominance of HMW *n*-alkanes (Fig. S1a) with the concentrations of LMW *n*-alkanes (242 ng m^−3^) being two times higher than HMW *n*-alkanes (111 ng m^−3^; Fig. 1a). In addition, the C_max_ values are obtained at C_23_ and C_25_ (Fig. 2a; Table S1), while C_23_ is the abundant homolog in most of the samples. CPI are used to identify pollution sources in aerosol^36^,^37^,^38^. CPI values obtained for C_19_ to C_36_ are 1.6 ± 0.35 in daytime and 1.4 ± 0.1 in nighttime (Table 1; Fig. 1a). They are in good agreement with those reported in Beijing and other urban aerosols from China (1.0 ± 0.43)^34^ but lower than those reported in mountain aerosols (up to 8.0)^2^ and marine aerosols (1.2–13)^13^,^27^. These results suggest that *n*-alkanes in Beijing were mainly derived from incomplete combustion of fossil fuels and petroleum residue.

#### Fatty acids

Fatty acids (C_8_–C_32_) including three unsaturated fatty acids (palmitoleic acid (C_16:1_), oleic acid (C_18:1_), and linoleic acid (C_18:2_)) are identified and quantified in the Beijing aerosol samples (Table S1). The molecular distribution of fatty acids is characterized by a strong even carbon number predominance with C_max_ at *n*-hexadecanoic acids (C_16:0_), followed by *n*-octadecanoic acids (C_18:0_) (Figs 2b and S1b). A similar pattern has been reported in the other urban aerosols in China^32^, India^17^ and USA^39^, but is different from the bimodal distribution observed for mountain aerosols^2^ or for marine aerosols, with maxima at C_16:0_ and C_24:0_/C_28:0_^35^,^40^.

The concentrations of fatty acids range from 137 to 3310 ng m^−3^ (871 ng m^−3^). Their averages were higher in nighttime (1010 ng m^–3^) than in daytime (733 ng m^–3^; Table 1; Fig. 1b) but not statistically significant (Table S2). There is no large difference between the concentrations of fatty acids in Beijing and other megacities in China (318–3240 ng m^−3^, 1070 ng m^−3^ in winter)^34^. However, these values are higher than those (302 ng m^−3^ in winter and 504 ng m^−3^ in summer) in Chennai aerosols^17^, and are 1–2 orders of magnitude higher than those (2.46–60.2 ng m^−3^, 13.8 ng m^−3^) in marine aerosols from the western north Pacific^35^.

Plant tissues consist of abundant fatty acids that can be emitted from biomass burning. While fossil fuel combustion, such as diesel truck exhaust and meat cooking, are also an important sources of fatty acids (C_16:0_, C_18:0_)^41^. Studies have demonstrated that the ratio of C_18:0_/C_16:0_ fatty acid can be used as a qualitative tool for source assessment^42^. The C_18:0_/C_16:0_ values are lower than 0.25 in aerosols from foliar vegetation combustion, waxy leaf surface abrasions, and wood smoke; values in the interval 0.25–0.5 are registered for car and diesel truck exhausts; values between 0.5 and 1.0 are achieved for cooking and paved and unpaved road dust^42^. The C_18:0_/C_16:0_ ratios in our study showed average values of 0.84 in the daytime versus 0.83 in the nighttime (Table 1), implying a strong input from cooking, road dust, and/or vehicle emissions for both C16:0 and C18:0 in Beijing.

Both biogenic and anthropogenic emissions are main sources of fatty acids in the atmosphere. High molecular weight (HMW, ≥C_20_) fatty acids are mostly derived from higher plants, while low molecular weight (LMW, <C_20:0_) fatty acids have multiple sources in urban aerosols, such as anthropogenic sources (such as kitchen emissions) or biomass combustion^8^,^32^,^35^,^36^,^37^,^38^,^39^,^40^,^41^,^42^,^43^. The average ratio of LMW/HMW is 12 in daytime versus 7.7 in nighttime (Table 1), which is much higher than mountain aerosols in China (1.0 ± 0.8)^2^ but similar to those (5.3 ± 1.8) reported in urban aerosols from Chennai, where the contribution of biomass burning is significant^17^. Furthermore, CPI values (2.4–7.6) of fatty acids imply that biogenic emission is also an important emitter of fatty acids in this region. Thus, these results demonstrate that fatty acids in Beijing aerosols can be explained by the mixed contributions of biogenic emission, biomass burning and/or anthropogenic source (such as cooking and/or vehicle emissions) in the urban area.

Unsaturated fatty acids are reported to emit directly from many sources such as the leaf surfaces of plants^12^, wood combustion^11^, meat charbroiling^9^, and marine biota^13^,^44^. Once unsaturated *n*-fatty acids are emitted into the atmosphere, the double bond in the structures can be oxidized, resulting in aldehydes and dicarboxylic acids^44^. Therefore, the concentration ratios between C_(18:0+16:0)_ and C_(18:n+16:1)_ (C_sat_/C_unsat_) can be used as a proxy to estimate the aging of organic aerosols in the atmosphere. The C_sat_/C_unsat_ ratios were 1.5–27 (14) in the daytime versus 1.6–20 (11) in the nighttime (Table 1). Although the difference between daytime and nighttime is insignificant (Table S2), slightly higher ratios of C_sat_/C_unsat_ still suggest an enhanced photochemical degradation of unsaturated fatty acids in the daytime^17^.

#### n-Alcohols

Normal fatty alcohols (C_16_–C_32_) were detected in the aerosol samples with a concentration range of 24.1–612 ng m^−3^ (109 ng m^−3^) in the daytime and 18.8–613 ng m^−3^ (190 ng m^−3^) in the nighttime (Tables 1 and S1), however the diurnal variations did not show statistical significance (Table S2). These results are slightly higher than those (6.1–527 ng m^−3^) reported in wintertime urban aerosols in fourteen Chinese megacities^34^. Compared with marine aerosols, the concentrations of *n*-alcohols in Beijing are one order of magnitude higher than those reported from remote Pacific and Atlantic Oceans aerosols (0.07–8.3 ng m^−3^)^40^. Again, higher concentrations of *n*-alcohols were observed in the nighttime, especially for C_21_–C_32_ species (Figs 1 and 2c). Simoneit^45^ reported that biomass burning processes can emit abundant fatty acids and *n*-alcohols into the air. The temporal patterns of *n*-alcohols are similar to that of fatty acids (Fig. 1b,c), suggesting that biomass-burning activities can enhance the emission of *n*-alcohols at a certain level.

The molecular distributions of *n-*alcohols were characterized by strong even-carbon numbered predominance (CPI = 1.9–10) with two maxima at C_26_ and C_28_ (Table 1 and Figs 2c and S1c). High molecular weight (HMW, ≥C_20_) *n*-alcohols are abundant in higher plant waxes and can also be emitted into the air by biomass burning^34^. Low molecular weight (LMW, <C_20_) *n*-alcohols are limited to soil microbes and marine biota^2^. The ratios of HMW/LMW ranged from 1.1 to 12 in the daytime and 0.97–16 in the nighttime (Table 1). These results suggest that higher plant waxes, biomass burning emissions and soil resuspension might be the sources of *n*-alcohols in this region, while their relative contributions varied with time.

### Stable carbon isotopic compositions of *n-*alkanes and fatty acids

The *δ*^13^C values of *n*-alkanes (C_20_–C_32_) and *n*-fatty acids (C_16:0_, C_18:0_, C_20:0_, C_22:0_, C_24:0_, C_26:0_, C_28:0_) were detected in Beijing aerosols. The *δ*^13^C values of individual compounds are provided in Tables S3 and S4 in the supporting information.

#### n-Alkanes

**Figure 3:**
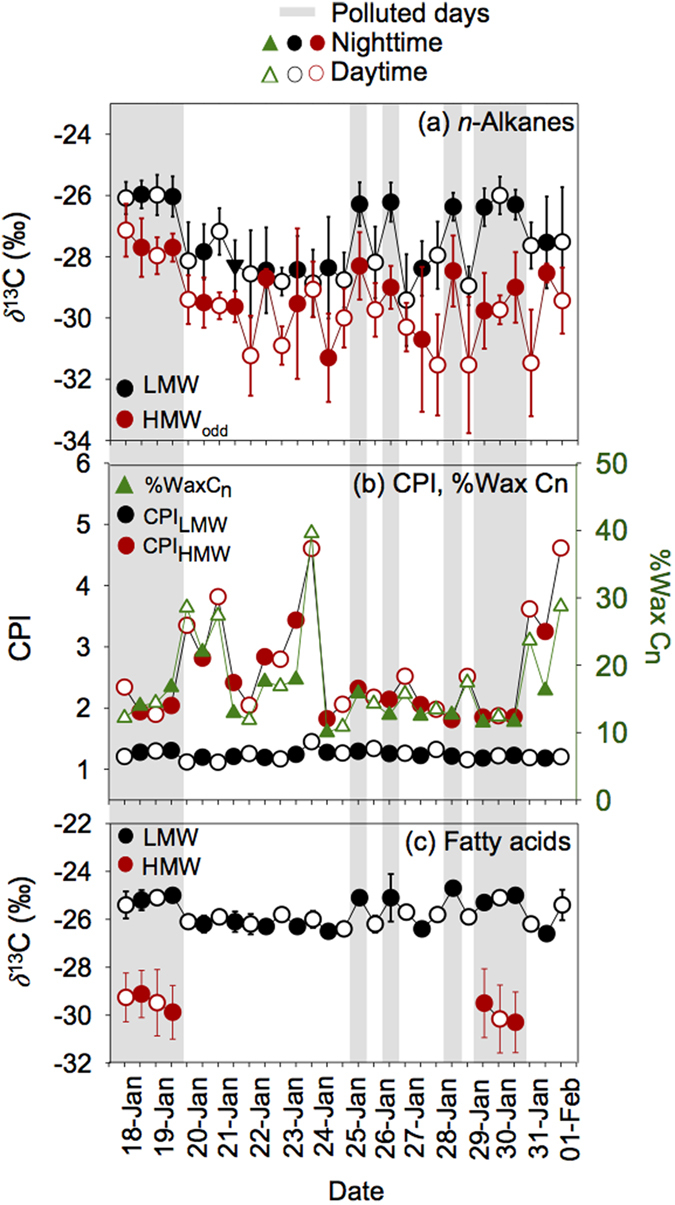
Temporal variations in the *δ*^13^C values and parameterizations of (**a**) *δ*^13^C values of LMW and HMW_odd_ *n*-alkanes, (**b**) CPI_LMW_,CPI_HMW_ and %Wax C_n_ of *n*-alkanes, (**c**) *δ*^13^C values of *n*-acids.

**Figure 4:**
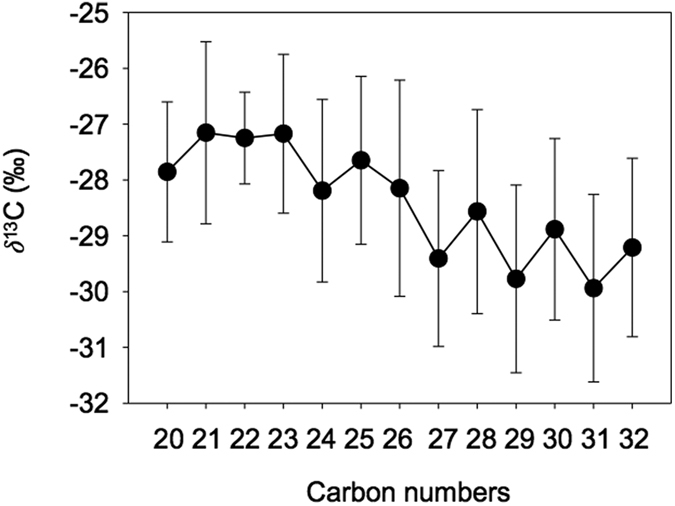
The *δ*^13^C distributions of individual *n*-alkanes throughout the sampling periods.

The *δ*^13^C values of homologous C_20_ to C_32_ *n*-alkanes detected in Beijing aerosols range from −34.1% to −24.7% (Table S3). In average, *δ*^13^C values in the nighttime (−27.9%) are slightly heavier than those in the daytime (−28.7%; Fig. 3a). The average *δ*^13^C values of individual lipids show a saw-tooth pattern (Fig. 4). The average *δ*^13^C ranges of C_20–25_ odd-carbon numbered *n*-alkanes (LMW_odd_: −27.3%) are slightly heavier than C_20–25_ even-carbon number homolog (LMW_even_: –27.7%); the average *δ*^13^C values of C_27–32_ odd-carbon numbered *n*-alkanes (HMW_odd_: –29.6%) are slightly depleted in ^13^C compared to C_27–32_ even-carbon number homolog (HMW_even_: −28.7%) (Fig. 4), exhibiting a similar pattern to those previously reported in urban aerosols from Japan^46^. Not all samples showed such saw tooth patterns, for example, samples with higher CPIs such as those collected in the daytime of 20 Jan, 24 Jan, and 1 Feb (Fig. S2). Meanwhile, the *δ*^13^C values of LMW_odd_ *n*-alkanes are highly constant (−25.6 ± 0.34%), suggesting a similar source for C_21_, C_23_ and C_25_.

**Figure 5:**
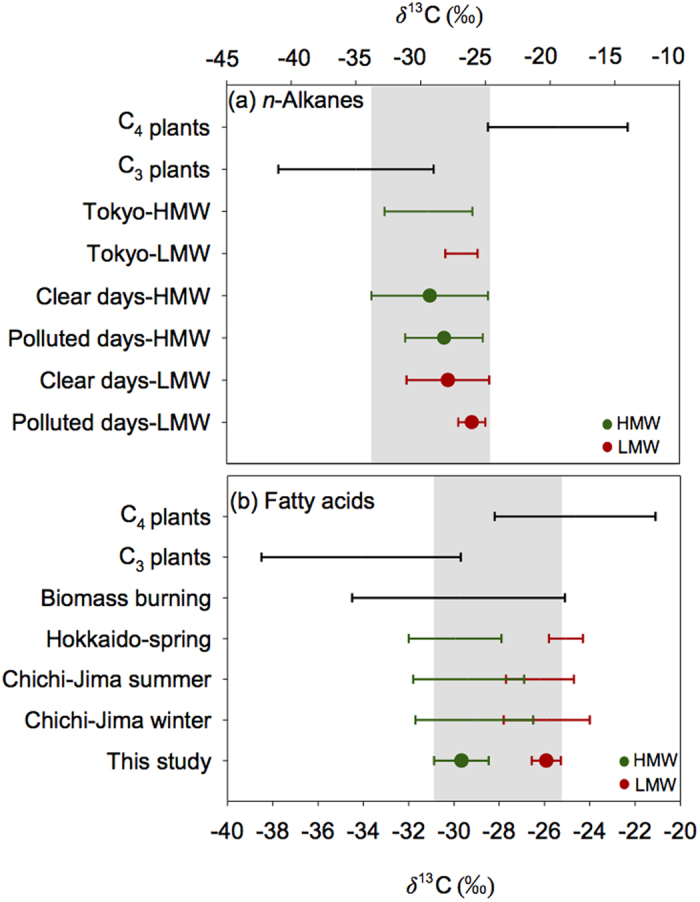
Comparison of the averaged *δ*^13^C ranges of (**a**) *n*-alkanes and (**b**) fatty acids between terrestrial higher plants, anthropogenic sources (such as vehicle exhaust and coal combustion) and urban aerosols from Beijing. The *δ*^13^C data of carbon sources of *n*-alkanes were reported for higher plants^26^,^49^,^50^, crude oil^54^,^55^, vehicle exhaust^56^, Tokyo^46^. The *δ*^13^C data of fatty acids were reported for higher plants^26^,^49^,^50^, Hokkaido^57^, and Chichi-Jima Island^26^.

LMW *n*-alkanes are the dominant species for most of the samples, while HMW_odd_ *n*-alkanes which are the typical lipid compounds derived from biogenic sources^47^ contribute less than LMW *n*-alkanes in this study (Fig. 1a). Average *δ*^13^C values of LMW *n*-alkanes were heavier than HMW_odd_ *n*-alkanes (Fig. 3a). The CPI_LMW_ (1.3; Fig. 3b) further demonstrate LMW *n*-alkanes are mainly from anthropogenic emissions, while the higher CPI_HMW_ (2.6) suggest that higher plants contribute more significantly for HMW_odd_ *n*-alkanes. Parts of the *δ*^13^C values of HMW *n*-alkanes fall into the data from C_3_ plants again demonstrate this point (Fig. 5a). In a previous study^46^, a positive correlation was found between δ^13^C values of HMW_odd_ *n*-alkanes against CPIs in urban aerosols from Tokyo (R^2^ = 0.5), while such a correlation was weak (R^2^ < 0.1 in Beijing). The reason for that is because higher plants contribute less than anthropogenic sources in wintertime Beijing aerosols. The percentage of wax *n*-alkanes (%Wax C_n_) is the contribution of biogenic *n*-alkane that is derived from higher plants waxes. There a linear relation (R^2^ ≥ 0.95, p < 0.01) between %Wax C_n_ and CPI (Fig. S3), which is consistent with that reported in other studies from China (R^2^ = 0.91)^48^ and Poland (R^2^ = 0.85)^37^, demonstrating that %Wax C_n_ here is the effective index to reflect the contribution of plant wax. The average contributions of biogenic *n*-alkanes to the total *n*-alkanes (%Wax C_n_) are 19% in daytime and 15% in nighttime, respectively (Table 1; Fig. 3b) suggesting minor input from leaf epicuticular waxes.

#### n-Fatty acids

*δ*^13^C values of C_16:0_ and C_18:0_ fatty acids were measured in all the samples. *δ*^13^C values of even-number fatty acids from C_20:0_ to C_28:0_ were only available for a few samples collected on polluted days (Fig. 3c; Table S4). *δ*^13^C values of C_16:0_ and C_18:0_ fatty acids range from −26.9% to −24.6% and are heavier than HMW fatty acids (−31.6% to −27.3%), suggesting the different sources. Fatty acids from plants with C_3_ and C_4_ photosynthetic pathways differ largely in the isotope fractionations^21^. Stable C isotope ratios of C_16:0_ and C_18:0_ fatty acids fall into biomass-burning aerosols (source material from C_3_ and C_4_ plant) that vary from –34.5% to −25.1%^49^,^50^ (Fig. 5b), further suggesting that biomass-burning emissions are the mainly source of LMW fatty acids. The *δ*^13^C values of HMW fatty acids have similar ranges compared with those from other urban aerosols that mainly influenced by higher plants; parts of *δ*^13^C values of HMW fatty acids also fall into C_3_ plants and biomass-burning aerosols, implying mixed sources of HMW fatty acids.

### Differences of atmospheric lipids between polluted and clear days

**Figure 6:**
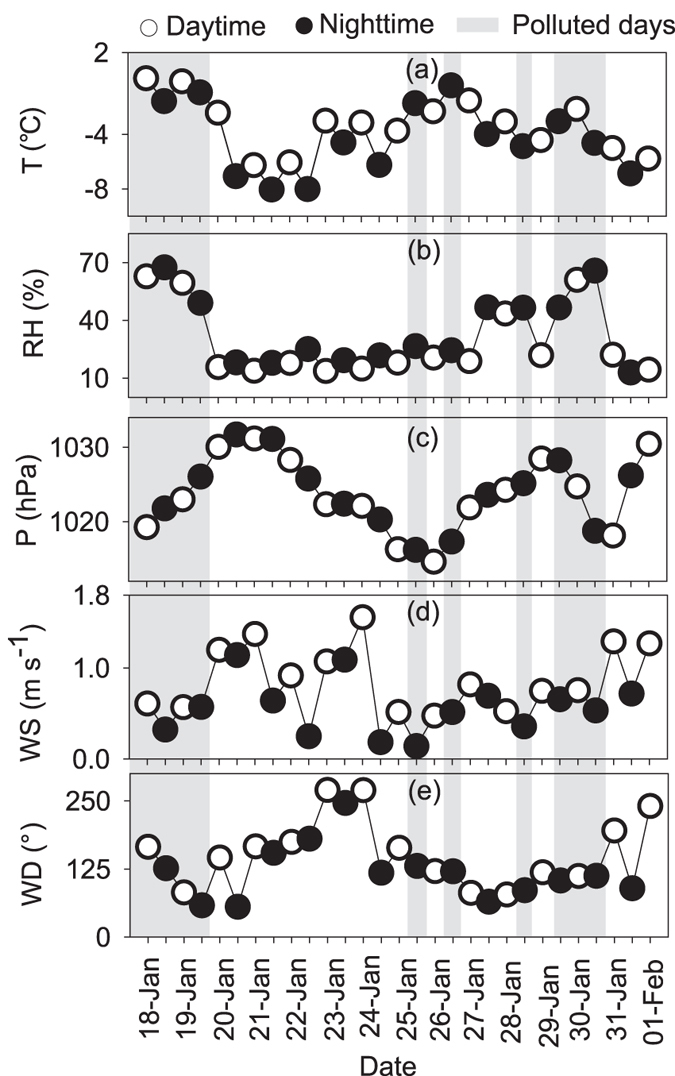
Time series of (**a**) temperature (T); (**b**) relative humidity (RH); (**c**) air pressure (P); (**d**) wind speed (WS); (**e)** wind direction (WD).

To investigate the variations during the pollution evolution process, the sampling period was divided into two categories according to monitoring results of the Beijing municipal environmental monitoring center: polluted and clear days. The differences between polluted and clear periods were estimated from the air pollution index (API) using the mass balance equation:


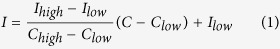


where I and C are the API and the concentration of PM_10_, respectively. C_high_ and C_low_ are values close to C, while I_high_ and I_low_ are values close to I (Table S5). Monitoring of pollutants consists of SO_2_, NO_2_, PM_10_, CO and O_3_. Figure 6 shows the temporal variations of the meteorological parameters (temperature (T; Fig. 6a), relative humidity (RH; Fig. 6b), air pressure (P; Fig. 6c), wind speed (WS; Fig. 6d) and wind direction (WD; Fig. 6e) to further explore the differences between polluted and clear days.

Concentrations of lipids in the nighttime are higher than those in the daytime (Figs 1 and 2); their atmospheric levels on polluted days are approximately six times higher than those on clear days and the differences are statistically significant (p < 0.01; Table S2; Fig. S4). Relative humidity on polluted days (51%) or in the nighttime (36%) are much higher than those on clear days (21%) or in the daytime (25%), respectively. RH can affects the aerosol formation in many ways such as aqueous-phase reactions and gas-particle partitioning and favor the formation of severe haze pollution episodes in Beijing^51^.

*δ*^13^C values of *n*-alkanes and fatty acids are heavier on polluted days than those on clear days. However, LMW lipids show a minor difference of *δ*^13^C values on polluted days, suggesting that they have a similar source during haze events (Fig. 3a,c). During the hazy days, air masses mainly originated from the south (Fig. S5), which were associated with relatively higher temperature, lower wind speed (0.5 m s^−1^), lower air pressure and lower heights of atmospheric boundary layer (Fig. 6). Such meteorological conditions made the ground convergence stronger, prevented the diffusion of atmospheric aerosols, leading to the narrow range of *δ*^13^C values of *n*-alkanes. Inversely, concentrations of lipids are very low and their *δ*^13^C values vary significantly on clear days (Figs 1, 2, 3). Results of back trajectories show that air masses are mainly from northwest Asian continent on clear days (Fig. S5). Clear air masses from northwestern regions via long-range atmospheric transport bring fresh aerosols and are likely to dilute and mix with local polluted aerosols, causing the complexity and mixed nature of aerosol particles in Beijing.

**Figure 7:**
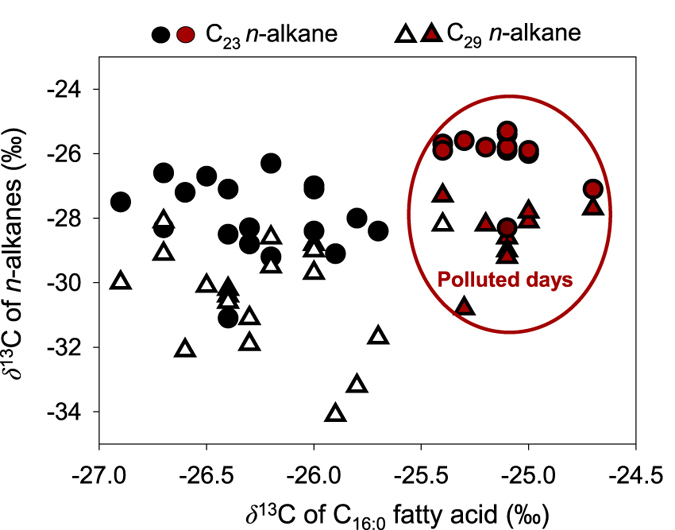
The correlations between the *δ*^13^C values of C_16:0_ fatty acid and *δ*^13^C values of *n*-alkanes.

C_23_ *n*-alkane is a representative marker component of anthropogenic origin^17^,^24^, while C_29_ *n*-alkane is a typical biomarker that originates from terrestrial higher plants^47^. We plot *δ*^13^C values of C_16:0_ fatty acid and C_23_ and C_29_ of *n*-alkanes to further assess the sources of Beijing aerosols. Figure 7 shows that the stable C isotope ratios on polluted days differ obviously from those on clear days, indicating that lipid compounds have different sources. However, *δ*^13^C values of *n*-alkanes are within the same range on polluted and clear days, while the values of fatty acids vary significantly with heavier values on polluted days. This suggests that *δ*^13^C value of individual fatty acids are a better proxy to investigate the sources of Beijing aerosols.

### Long-range atmospheric transport of aerosol particles

The reactivity of the unsaturated fatty acids can be used to determine the source and photochemical aging of atmospheric aerosols^52^. For example, compared with fatty acids derived from local emissions, aged aerosols suffer from photochemical degradation during long-range transport and thus have lower contents of unsaturated fatty acids, which result in a high ratio of C_sat_/C_unsat_. In our study, C_sat_/C_unsat_ ratios on polluted and clear days were 7.2 and 14, respectively. These results provide evidence that aerosol particles were more aged through long-distance transport on clear days (Fig. S5). In contrast, fatty acids were mainly derived from local biological, anthropogenic sources such as cooking, transportation and/or biomass burning on polluted days. This is consistent with air mass back trajectories that they were most likely influenced by local emissions on polluted days and originated from the long-range atmospheric transport of terrestrial aerosols from the northern regions of the continent on clear days (Fig. S5).

**Figure 8:**
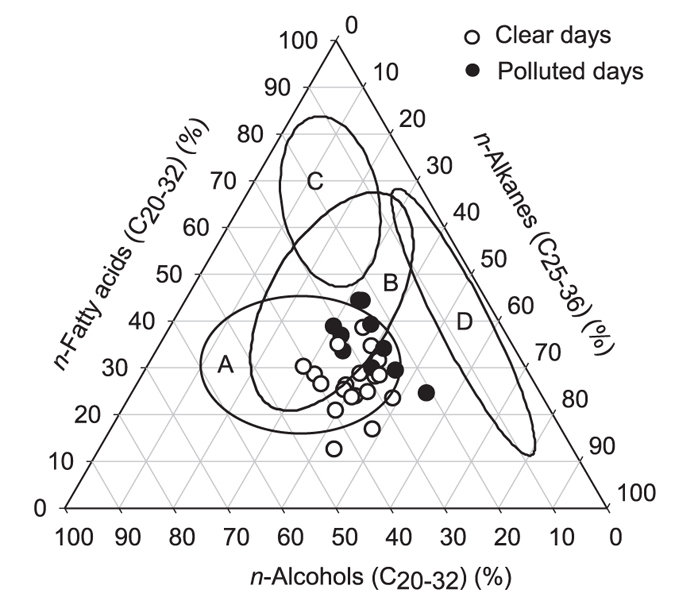
Triangular plots of relative abundances of terrestrial biomarkers. *n*-alkanes (C_25_–C_36_), fatty acids (C_20_–C_32_) and fatty alcohols (C_20_–C_32_) in the aerosol samples collected from Beijing. The mountain aerosols collected at Mt. Tai, North China Plain fall in category A^2^. The marine samples collected from Chichi-Jima Island fall into category B (during winter/spring seasons) and category C (during summer/autumn seasons)^35^, while the marine samples from the western Pacific^23^ fall into category D.

Furthermore, we plot the relative abundances of *n*-alkanes (C_25–36_), *n*-fatty acids (C_20–32_) and *n*-alcohols (C_20–32_), the typical terrestrial biomarkers, in a triangular diagram (Fig. 8). The results show that the aerosol samples on polluted days have higher *n*-alkanes (32–54%) and/or *n*-fatty acids (25–44%), while higher abundant *n*-alcohols (26–44%) occurred on clear days. The distribution of these plots in Beijing is different from other aerosol samples from mountain (category A) and marine regions (category B, C, D), which indicates that the main origins of terrestrial organic matter are probably different. Most of the aerosol samples fall in a relatively narrow range, being similar to those of the mountain aerosols collected at the summit of Mt. Tai in the North China Plain during a severe biomass-burning season (category A)^2^, which suggests that biomass-burning emission might be the significant source of terrestrial biomarkers in Beijing aerosols in wintertime. The data from Beijing aerosols are plotted in category B, which were collected under the condition of westerly winds from the Asian continent, indicating that the similarities in the distribution are more likely attributed to an identical source. The Asian continent should be one of the major source regions for terrestrial biomarkers transported over the western Pacific. Although the data from the summer and autumn seasons (category C) and the data from the western Pacific (category D) did not include the samples from Beijing aerosols, categories C and D are much closer to the urban aerosols, which again indicates that Asian aerosols are possibly one of the important sources of terrestrial organic matter in the western Pacific.

The *δ*^13^C values of *n*-alkanes and *n*-fatty acids again support such a point. LMW *n*-alkanes have different *δ*^13^C values with HMW_odd_ *n*-alkanes on polluted days, which are consistent with fatty acids (Fig. 3a,c), suggesting although experienced similar atmospheric pathways and transport processes over the western Pacific, whereas the difference in *δ*^13^C reflects the different sources of the lipids. As mentioned above, LMW *n*-alkanes and *n*-acids on polluted days are mainly originated from anthropogenic source associated with southern air masses. While most of the *δ*^13^C values of LMW *n*-alkanes and *n*-acids detected in Beijing aerosols fall into the downwind urban and marine aerosols^26^ (Fig. 5) which were influenced by the Asian continental aerosols through long-range atmospheric transport.

## Materials and Methods

### Aerosol sampling

Aerosol sampling was performed on the roof-top of a three-story building (10 m above ground level) at the Institute of Atmospheric Physics, Chinese Academy of Sciences (39°58′28″N, 116°22′13″E), Beijing, which is located in the northern part of the northern China plain. Total suspended particle (TSP) samples (n = 29) were collected on a 12-hour basis from 18 January to 1 February 2012. These samples were collected using pre-combusted (6 h in 450 °C in a muffle furnace) quartz filters (20 cm × 25 cm, Pallflex) and a high-volume air sampler at a flow rate of 1 m^3^_ _min^−1^. After the sampling, the filters were stored in a pre-combusted glass jar with a Teflon-lined screw cap in a freezer at −20 °C until analysis.

### Measurements of lipids

An aliquot of filter (ca. 25 cm^2^) sample was extracted with dichloromethane (DCM)/methanol (MeOH) (2:1, *v*/*v*) for three times under ultrasonication for 10 min each. After filtering through Pasteur pipette packing with quartz wool, the supernatant was concentrated with a rotary evaporator and then blown down to dryness with pure nitrogen gas. The extracts were reacted with 50 μl of N,O-bis-(trimethylsilyl) trifluoroacetamide (BSTFA) with 1% trimethylsilyl chloride and 10 μl of pyridine at 70 °C for 3 h to derive trimethylsilyl derivatives. After the derivation, the solution was diluted with 140 μl of *n*-hexane containing the internal standard (C_13_ *n*-alkane, 1.43 ng μl^–1^) and preserved in a freezer (−20 °C) until GC/MS analysis.

Lipid class compounds (*n*-alkanes, fatty acids and *n*-alcohols) were measured using a Hewlett-Packard model 6890 GC coupled with a Hewlett-Packard model 5973 mass-selective detector (MSD). The GC is equipped with an on-column injector and a DB-5 MS fused silica capillary column (30 m × 0.25 mm, 0.25 μm film thickness). The GC oven temperature was held at 50 °C for 2 min, programmed from 50 to 120 °C at 30 °C min^–1^ followed by 120 to 300 °C at a rate of 6 °C min^–1^ and then held isothermally at 300 °C for 16 min. GC/MS data were processed by the Chemstation software. Recoveries for the authentic standards of lipid compounds that were spiked into pre-combusted quartz filters were better than 80%. The relative standard deviation of the concentrations based on duplicate analysis was <15%.

### Determination of compound-specific stable carbon isotopic compositions

Prior to the measurement of the ^13^C/^12^C ratios of *n*-alkanes and fatty acids, filter samples were extracted with DCM)/MeOH (2:1, *v*/*v*) for three times. The extracts were then passed through LC-NH_2_ solid phase extraction (SPE) tubes. The neutral and acid fractions of the lipid extract adsorbed on to the LC-NH_2_ SPE tube were eluted with DCM/isopropyl alcohol (2:1, *v/v*) and ether with 4% acetic acid, respectively, and collected into separate sample vials and dried under nitrogen gas blow. The fraction of *n*-alkanes was then separated from the neutral fraction of the extract on a silica gel column with hexane and preserved in a freezer (−20 °C) until analysis. To separate the fatty acids from the acid fraction of the extract, 0.3 ml of toluene and 1 ml of acidified methanol were added to the sample vial and blow down with nitrogen for 5 s and then tightly capped in the vial. The sample vial was then heated at 60 °C for 12 hrs followed by cooling to room temperature. Then, 5 ml of organic free NaCl (5%) water, 1 ml of hexane were added to the sample vial, and the solvent fraction was extracted into another sample vial with a pipette. The solvent extracts were concentrated, and then the fatty acids were separated using a small silica gel column with hexane and preserved in a freezer (−20 °C) until analysis.

*δ*^13^C of individual *n*-alkanes (and fatty acids) were determined using a Thermo Trace GC Ultra coupled with a Gas Isotope Ratio MS (MAT 253) via a combustion furnace maintained at a temperature of 1000 °C. The GC was equipped with an on-column injector and a DB-5 MS fused silica capillary column (30 m × 0.25 mm, 0.25 μm film thickness). The GC oven temperature was held at 50 °C for 2 min, programmed from 50 to 120 °C at 30 °C min^–1^ followed by 120 to 300 °C at a rate of 6 °C min^–1^ and held isothermally at 300 °C for 16 min^–1^.

*δ*^13^C of individual fatty acid was estimated from the isotopic signature of the methyl esters using the mass balance equation:


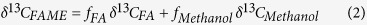


where C_Methanol_, *δ*^13^C_FA_, *δ*^13^C_FAME_ are the carbon isotope signatures of the methanol, the underivatized fatty acid, and the fatty acid methyl esters, respectively. f_Methanol_ and f_FA_ are the fractions of carbon in the fatty acid methyl esters due to the methanol and the underivatized fatty acid, respectively^26^. The isotope ratios of individual fatty acids were calculated using the *δ*^13^C_Methanol_ value of −21.1‰. The analytical error of the δ^13^C values of each compound based on duplicate analysis was <0.4‰.

## Additional Information

**How to cite this article**: Ren, L. *et al.* Molecular distributions and compound-specific stable carbon isotopic compositions of lipids in wintertime aerosols from Beijing. *Sci. Rep.* **6**, 27481; doi: 10.1038/srep27481 (2016).

## Supplementary Material

Supplementary Information

## Figures and Tables

**Table 1:** Concentrations and other parameters of lipid compounds in aerosol samples (TSP) collected in Beijing.

	Whole period			Daytime (n = 15)			Nighttime (n = 14)		
	Range	Mean	SD	Range	Mean	SD	Range	Mean	SD
I. *n*-Alkanes									
Conc. (ng m^−3^)	40.1–1720	367	447	40.1–1440	262	363	55.3–1720	472	514
CPI^a^	1.3–2.5	1.5	0.28	1.3–2.5	1.6	0.35	1.3–1.6	1.4	0.1
CPI_LMW_	1.1–1.5	1.3	0.06	1.1–1.5	1.2	0.08	1.2–1.3	1.2	0.04
CPI_HMW_	1.3–4.7	2.6	0.81	1.8–4.7	2.8	0.95	1.3–3.4	2.3	0.54
%Wax C_n_^b^	10–40	17	0.07	11–40	19	0.08	10–22	15	0.03
LMW/HMW	0.94–4.5	2.3	0.76	0.94–3.4	2.1	0.82	1.7–4.5	2.5	0.67
II. Fatty acids									
Conc. (ng m^−3^)	137–3306	871	871	187–3310	733	783	137–2120	1010	966
CPI^a^	2.4–7.6	4.8	1.4	2.4–7.6	5.0	1.6	3.1–6.9	4.7	1.1
C_18:0_/C_16:0_	0.5–1.28	0.83	0.24	0.53–1.19	0.84	0.24	0.5–1.28	0.83	0.25
C_sat_/C_unsat_	1.5–27	12	0.23	1.5–27	14	0.21	1.6–20	11	0.25
LMW/HMW	1.0–39	10	8.1	2.6–39	12	10	1.0–17	7.7	4.6
III. *n*-Alcohols									
Conc. (ng m^−3^)	18.8–613	149	174	24.1–612	109	147	18.8–613	190	205
CPI^a^	1.9–10	4.5	2.0	1.9–8.3	4.1	1.6	2.4–10	4.8	2.5
HMW/LMW	0.97–16	5.5	3.3	1.1–12	5.0	2.6	0.97–16	6.0	3.8

^a^CPI, carbon preference index, Σodd (C_21–35_)/Σeven (C_20–36_) for *n*-alkanes; Σeven (C_20–32_)/Σodd (C_21–31_) for fatty acids; Σeven (C_20–32_)/Σodd (C_21–31)_ for *n*-alcohols.

^b^ %WaxC_n_ = Σ(C_n _− 0.5(C_n+1_ + C_n−1_))/ΣC_n _× 100%. Negative values of C_n_ were taken as zero.
