# Supplementary material for: Molecular distributions and compound-specific stable carbon isotopic compositions of lipids in wintertime aerosols from Beijing
Source: Sci Rep. 2016 Jun 8;6:27481. doi: 10.1038/srep27481 (PMC4897684; doi:10.1038/srep27481)
Supplement: Supplementary Information [file srep27481-s1.pdf]

Supplementary Information

Molecular distributions and compound-specific stable carbon isotopic compositions of lipids in wintertime aerosols from Beijing

Lujie Ren[1,6], Pingqing Fu[1,2*], Yue He[3], Juzhi Hou[3], Jing Chen[4], Chandra Mouli Pavuluri[5], Yele Sun[1] & Zifa Wang[1]

[1]State Key Laboratory of Atmospheric Boundary Layer Physics and Atmospheric Chemistry, Institute of Atmospheric Physics, Chinese Academy of Sciences, Beijing 100029, China

[2]Collaborative Innovation Center on Forecast and Evaluation of Meteorological Disasters, Nanjing University of Information Science & Technology, Nanjing, 210044, China

[3]Institute of Tibetan Plateau Research, Chinese Academy of Sciences, Beijing 100085, China

[4]Institute of Geographic Sciences and Natural Resources Research, Chinese Academy of Sciences, Beijing 100101, China

[5]Institute of Surface-Earth System Science, Tianjin University, Tianjin 300072, China

[6]University of Chinese Academy of Sciences, Beijing 100049, China

*Corresponding author email: fupingqing@mail.iap.ac.cn; Phone: +86-10-8201-3200

This file contains five tables (Table S1-S5) and five figures (Figure S1-S5).

Table S1. Concentrations (ng m^{-3}) of individual lipids compounds in the urban aerosol samples

	Whole period	Daytime (n=15)			Nighttime (n=14)		
	Range	Range	Mean	SD	Range	Mean	SD
***n*-Alkanes**							
C_{19}	2.36–26.5	2.36–26.5	7.95	6.82	3.05–25.8	11.6	6.67
C_{20}	3.18–84.9	3.18–57.7	13.6	16.4	4.83–84.9	28.7	23.1
C_{21}	3.68–173	3.68–141	23.5	35.6	5.67–173	49.1	49.4
C_{22}	2.86–210	2.86–175	25.8	44.5	6.15–210	55.6	60.8
C_{23}	3.88–242	3.88–201	29.6	51.7	6.99–242	63.7	70.6
C_{24}	2.81–184	2.81–184	25.5	47.2	5.87–211	56.4	62.9
C_{25}	3.56–224	3.56–185	25.7	47.3	6.14–224	59.4	66.8
C_{26}	1.95–135	1.95–110	15.3	28.1	3.49–135	34.6	40.3
C_{27}	3.22–125	3.22–102	15.2	25.6	3.49–125	32.3	38.8
C_{28}	0.76–54	0.76–46.1	6.23	11.6	1.15–54.0	13.7	16.9
C_{29}	3.35–103	5.10–89.3	14.4	21.6	3.35–103	26.8	33.2
C_{30}	0.5–29.5	0.76–26.1	3.57	6.38	0.50–29.5	7.87	9.68
C_{31}	2.58–57.8	2.94–43.1	7.50	10.1	2.58–57.8	14.6	17.1
C_{32}	0.29–21.8	0.29–19.0	2.48	4.63	0.39–21.8	4.99	6.68
C_{33}	0.38–27.8	0.38–21.3	2.97	5.16	0.51–27.8	6.71	8.43
C_{34}	0.11–10.4	0.18–8.5	1.12	2.06	0.11–10.4	2.29	3.21
C_{35}	0.09–10.8	0.15–10.2	1.15	2.52	0.09–10.8	2.28	3.25
C_{36}	0–7.9	0–5.49	0.42	1.41	0–7.9	1.25	2.27
subtotal	40.1–1720	40.1–1440	262	363	55.3–1720	472	514
CPI	1.29–2.54	1.31–2.54	1.57	0.35	1.29–1.6	1.38	0.1
%Wax Cn	10–39.6	10.9–39.6	19.2	0.08	10–22	14.6	0.03
**Fatty acids**							
$C_{8:0}$	1.77–10.1	2.51–10.1	4.31	1.85	1.77–9.44	1.90	4.79
$C_{9:0}$	3.26–18.0	3.37–11.0	7.08	2.27	3.26–18.0	4.01	8.12
$C_{10:0}$	0.97–13.4	1.03–9.87	2.96	2.37	0.97–13.4	3.26	4.27
$C_{11:0}$	0.51–15.8	0.42–13.9	2.32	3.34	0.50–15.8	5.03	4.69
$C_{12:0}$	1.58–66.1	3.1–42.7	10.5	14.1	1.58–66.1	19.1	15.6
$C_{13:0}$	0.19–10.1	0.24–8.67	1.28	2.09	0.19–10.1	2.98	2.20
$C_{14:0}$	4.26–182	6.51–118	19.3	27.8	4.26–182	46.3	33.9
$C_{15:0}$	1.27–25.6	1.19–31.1	5.38	7.38	1.27–25.6	9.32	10.2
$C_{16:0}$	65.3–1050	96–838	240	199	65.3–1050	271	315
$C_{17:0}$	1.15–75.6	0.98–72.1	12.5	23.6	1.15–75.6	21.2	18.5
$C_{18:0}$	37.7–1270	55.7–999	208	255	37.7–1268	346	261
$C_{19:0}$	0.35–28.8	0.26–28.8	3.32	7.10	0.35–21.4	7.02	6.25
$C_{20:0}$	1.35–118	1.35–118	14.5	28.8	2.63–88.2	32.7	29.2
$C_{21:0}$	0.28–41.0	0.28–34.4	3.83	8.54	0.42–41.1	12.3	8.85

$C_{22:0}$	0.84–132	0.84–127	19.2	40.3	1.10–132	42.5	34.3
$C_{23:0}$	0.20–82.8	0.55–73.4	6.93	18.5	0.20–82.8	25.1	15.5
$C_{24:0}$	1.01–181	1.01–167	17.68	41.79	1.20–181	59.4	41.8
$C_{25:0}$	0.16–49.9	0.22–45.1	4.19	11.3	0.16–49.9	15.1	9.43
$C_{26:0}$	0.99–177	0.55–148	14.6	36.9	0.99–177	56.0	36.1
$C_{27:0}$	0.19–31.1	0.21–26.1	2.51	6.51	0.19–31.0	8.96	4.95
$C_{28:0}$	0.49–109	0.20–95.6	9.17	24.0	0.49–109	34.1	21.5
$C_{29:0}$	0–19.0	0–16.6	1.56	4.17	0–19.0	5.45	3.06
$C_{30:0}$	0–66.9	0–54.6	4.97	13.73	0–66.9	20.6	11.1
$C_{31:0}$	0–5.44	0–5.44	0.36	1.40	0–5.11	1.50	0.62
$C_{32:0}$	0–17.0	0–13.3	0.89	3.44	0–17.1	4.94	1.93
$C_{16:1}$	1.19–73.8	1.19–44.1	9.04	13.1	1.35–73.8	21.0	13.9
$C_{18:1}$	6.14–275	6.14–142	34.26	43.3	6.16–275	78.7	62.4
$C_{18:2}$	0.36–90.8	0.36–60.4	9.90	16.1	0.46–90.8	25.5	15.0
subtotal	137–3306	187–3310	733	783	137–2120	1010	966
CPI	2.37–7.58	2.37–7.58	5.01	1.62	3.11–6.89	4.68	1.07
$C_{18:0}/C_{16:0}$	0.5–1.28	0.53–1.19	0.84	0.24	0.5–1.28	0.83	0.25
$C_{18:n}/C_{16:0}$	1.52–27.4	1.52–27.4	14.2	0.21	1.59–19.8	10.6	0.25
***n*-Alcohols**							
C_{16}	0.67–13.7	0.82–11.2	2.87	3.66	0.67–13.7	13.7	4.61
C_{17}	0.50–12.4	0.50–12.4	2.08	3.34	0.58–7.6	7.6	2.35
C_{18}	1.31–191	1.58–94.6	13.6	24.0	1.31–191	191	25.4
C_{19}	0.32–17.3	0.39–9.47	2.36	2.62	0.32–17.3	17.3	4.41
C_{20}	0.54–28.7	0.54–19.0	3.31	4.50	0.87–28.7	28.7	7.82
C_{21}	0.57–12.3	0.69–12.3	1.96	3.08	0.57–11.5	11.5	3.89
C_{22}	1.04–77.2	1.12–66.6	8.02	16.4	1.04–77.2	77.2	17.6
C_{23}	0.47–19.5	0.89–19.5	3.26	5.28	0.47–15.5	15.5	4.74
C_{24}	2.19–42.3	2.31–41.7	6.81	11.2	2.19–42.3	42.3	13.6
C_{25}	0.40–12.0	0.71–12.0	2.11	3.77	0.40–10.9	10.9	3.36
C_{26}	3.34–108	3.51–80.4	13.4	18.8	3.34–108	108	26.7
C_{27}	0.53–24.5	0.72–21.7	2.89	5.31	0.53–24.5	24.5	4.81
C_{28}	3.26–133	3.92–93.0	14.9	21.8	3.26–133	133	31.7
C_{29}	0.24–29.4	0.24–29.4	3.39	7.35	0.53–18.8	18.8	4.51
C_{30}	0–101	2.43–70.4	11.2	17.7	0.90–101	101	25.4
C_{31}	0–7.9	0–7.27	1.52	2.18	0–7.9	7.9	1.79
C_{32}	0–33.4	0–24.3	3.09	5.94	0–33.4	33.4	6.91
subtotal	18.8–613	24.1–612	109	147	18.8–613	190	205
CPI[b]	1.85–10.3	1.85–8.29	4.1	1.6	2.44–10.3	4.84	2.5
HMW/LMW	0.97–15.5	1.07–11.6	5	2.6	0.97–15.5	5.99	3.8

Table S2. Independent samples test (t-test) of lipids between nighttime and daytime, polluted days and clear days.

		Levene′s Test for Equality of Variance		T-test for Equality of Means		
		F	Sig	t	df	Sig.(2-tailed)
n-Alkanes[a] (ng m^{-3})	Equal variance assumed	1.344	0.256	-1.277	27	0.213
Fatty acids[a] (ng m^{-3})	Equal variance assumed	2.290	0.142	-0.794	27	0.434
n-Alcohols[a] (ng m^{-3})	Equal variance assumed	0.289	0.104	-1.05	27	0.302
Total lipids[a] (ng m^{-3})	Equal variance assumed	2.839	0.104	-1.05	27	0.302
C_{sat}/C_{unsat}[a]	Equal variance assumed	1.156	0.292	-0.045	27	0.964
Total lipids[b] (ng m^{-3})	Equal variance assumed	30.2	0.000	5.42	27	0.000
	Equal variance not assumed			4.27	10.5	0.001

[a] T-test results between nighttime and daytime;

[b] T-test results between polluted days and clear days.

Table S3. Compound-specific stable carbon isotope ratios of *n*-alkanes in the Beijing aerosols collected in January 2012.

Date	CPI	C_{20}	C_{21}	C_{22}	C_{23}	C_{24}	C_{25}	C_{26}	C_{27}	C_{28}	C_{29}	C_{30}	C_{31}	C_{32}
18 Jan.D[a]	1.31	-25.9	-25.8	-26.7	-25.7	-26.8	-25.6	-25.7	-26.2	-25.2	-27.3	-25.8	-27.9	-27.6
18 Jan.N[b]	1.33	-26.1	-25.9	-26.5	-25.8	-26.3	-25.2	-27.8	-26.6	-25.6	-28.2	-26.9	-28.3	-26.9
19 Jan.D	1.32	-26.8	-26.1	-26.7	-25.4	-25.6	-25.3	-26.6	-27.4	-26.4	-28.6	-27.5	-27.9	-27.5
19 Jan.N	1.40	-26.7	-25.0	-26.8	-26.0	-25.8	-25.9	-27.0	-27.2	-26.2	-28.1	-27.3	-27.8	-27.5
20 Jan.D	1.88	-28.9	-28.6	-25.9	-27.5	-28.5	-29.4	-29.1	-29.7	-29.7	-30.0	-30.8	-28.5	-30.3
20 Jan.N	1.60	-27.7	-29.1	-26.8	-27.0	-27.9	-28.6	-25.7	-29.3	-27.3	-28.8	-26.2	-30.4	-30.7
21 Jan.D	1.84	-27.5	-28.1	-26.3	-26.6	-26.7	-27.9	-28.2	-29.9	-29.9	-29.1	-30.2	-29.8	-30.1
21 Jan.N	1.34	-27.7	-29.5	-27.4	-28.4	-28.9	-27.8	-24.8	-29.1	-28.9	-29.7	-27.9	-30.1	-27.0
22 Jan.D	1.32	-28.7	-29.4	-26.7	-27.1	-30.4	-29.1	-26.0	-30.8	-30.8	-30.2	-30.9	-32.7	-26.6
22 Jan.N	1.48	-29.6	-29.4	-27.2	-27.1	-30.1	-27.3	-28.1	-28.7	-28.7	-29.0	-29.1	-28.4	-26.7
23 Jan.D	1.45	-28.3	-29.0	-28.6	-28.8	-29.6	-28.6	-25.4	-30.2	-30.2	-31.1	-26.7	-31.4	-32.0
23 Jan.N	1.50	-29.7	-28.1	-27.2	-28.3	-29.8	-27.5	-26.3	-29.7	-27.7	-31.9	-28.8	-27.0	-27.2
24 Jan.D	2.54	-29.5	-27.1	-28.6	-28.3	-29.7	-30.0	-26.9	-29.9	-29.9	-28.1	-30.6	-29.2	-30.0
24 Jan.N	1.27	-27.5	-30.2	-26.7	-26.7	-30.3	-28.8	-28.7	-30.9	-26.9	-30.1	-30.2	-32.9	-28.6
25 Jan.D	1.28	-29.6	-28.5	-28.0	-28.5	-30.1	-27.9	-28.5	-30.7	-27.7	-30.4	-28.1	-28.9	-29.5
25 Jan.N	1.42	-26.6	-25.0	-26.9	-25.9	-26.7	-26.6	-28.3	-27.2	-28.1	-28.3	-30.1	-29.4	-31.2
26 Jan.D	1.36	-27.5	-27.5	-28.2	-29.2	-29.9	-26.8	-28.7	-29.0	-29.0	-29.5	-29.7	-30.7	-30.7
26 Jan.N	1.32	-26.8	-25.2	-26.9	-25.8	-26.3	-26.3	-28.0	-28.3	-28.3	-29.0	-29.1	-29.7	-30.7
27 Jan.D	1.43	-29.8	-26.8	-28.7	-31.1	-29.9	-30.2	-30.2	-30.9	-25.6	-30.6	-27.0	-29.4	-28.7
27 Jan.N	1.32	-29.2	-26.8	-28.0	-28.4	-28.9	-29.0	-29.9	-28.0	-28.0	-31.7	-29.9	-32.4	-31.1
28 Jan.D	1.34	-28.7	-26.3	-27.4	-28.0	-29.5	-27.8	-27.1	-31.5	-31.5	-33.2	-27.8	-29.9	-30.1
28 Jan.N	1.31	-26.2	-26.5	-26.4	-27.1	-26.3	-25.7	-27.9	-27.9	-27.9	-27.7	-29.7	-29.8	-30.6
29 Jan.D	1.46	-28.3	-28.9	-28.5	-29.1	-30.1	-28.8	-33.7	-30.4	-30.4	-34.1	-32.2	-30.1	-29.2
29 Jan.N	1.28	-26.7	-25.6	-26.9	-25.6	-26.9	-26.6	-28.5	-28.4	-28.4	-30.8	-28.0	-30.1	-30.8
30 Jan.D	1.31	-26.3	-25.2	-26.7	-25.3	-26.4	-26.1	-30.5	-30.1	-30.1	-29.2	-28.2	-29.9	-29.5
30 Jan.N	1.29	-26.1	-25.8	-26.9	-25.9	-26.2	-26.9	-30.0	-29.1	-29.1	-27.8	-28.9	-30.1	-28.6
31 Jan.D	1.7	-28.3	-26.9	-28.5	-27.2	-28.1	-26.8	-29.0	-32.8	-32.8	-32.1	-30.1	-29.5	-28.6
32 Jan.N	1.5	-28.4	-26.5	-26.4	-26.3	-27.5	-30.1	-28.4	-28.2	-28.2	-28.6	-28.6	-28.8	-28.1
01 Feb.D	1.94	-28.7	-24.7	-28.2	-25.9	-28.4	-29.2	-31.3	-29.9	-29.9	-28.2	-31.3	-30.2	-31.0

[a] D: The samples were collected in daytime.

[b] N: The samples were collected in nighttime.

Table S4. Stable individual carbon isotope of Fatty acids collected from Beijing aerosols in 2012.

	CPI	C_{16}	C_{18}	C_{20}	C_{22}	C_{24}	C_{26}	C_{28}
18 Jan.D[a]	1.31	-25.4	-26.2	-28.5	-28.8	-29.3	-28.7	-31.0
18 Jan.N[b]	1.33	-25.2	-24.6	-27.9	-29.2	-28.7	-29.2	-30.6
19 Jan.D	1.32	-25.1	-25.0	-28.3	-28.4	-29.0	-30.1	-31.5
19 Jan.N	1.40	-25.0	-24.9	-28.5	-30.0	-29.8	-29.5	-31.6
20 Jan.D	1.88	-26.1	-26.3	n.d	n.d	n.d	n.d	n.d
20 Jan.N	1.60	-26.2	-26.7	n.d	n.d	n.d	n.d	n.d
21 Jan.D	1.84	-25.9	-26.2	n.d	n.d	n.d	n.d	n.d
21 Jan.N	1.34	-26.1	-26.7	n.d	n.d	n.d	n.d	n.d
22 Jan.D	1.32	-26.2	-25.6	n.d	n.d	n.d	n.d	n.d
22 Jan.N	1.48	-26.3	-26.1	n.d	n.d	n.d	n.d	n.d
23 Jan.D	1.45	-25.8	-26.0	n.d	n.d	n.d	n.d	n.d
23 Jan.N	1.50	-26.3	-26.2	n.d	n.d	n.d	n.d	n.d
24 Jan.D	2.54	-26.0	-26.5	n.d	n.d	n.d	n.d	n.d
24 Jan.N	1.27	-26.5	-26.9	n.d	n.d	n.d	n.d	n.d
25 Jan.D	1.28	-26.4	-26.2	n.d	n.d	n.d	n.d	n.d
25 Jan.N	1.42	-25.1	-25.4	n.d	n.d	n.d	n.d	n.d
26 Jan.D	1.36	-26.2	-25.7	n.d	n.d	n.d	n.d	n.d
26 Jan.N	1.32	-25.1	-26.5	n.d	n.d	n.d	n.d	n.d
27 Jan.D	1.43	-25.7	-26.1	n.d	n.d	n.d	n.d	n.d
27 Jan.N	1.32	-26.4	-26.4	n.d	n.d	n.d	n.d	n.d
28 Jan.D	1.34	-25.8	-26.1	n.d	n.d	n.d	n.d	n.d
28 Jan.N	1.31	-24.7	-25.0	n.d	n.d	n.d	n.d	n.d
29 Jan.D	1.46	-25.9	-26.3	n.d	n.d	n.d	n.d	n.d
29 Jan.N	1.28	-25.3	-25.0	-27.3	-29.9	-29.6	-29.4	-31.3
30 Jan.D	1.31	-25.1	-25.1	-27.8	-30.7	-30.5	-30.2	-31.6
30 Jan.N	1.29	-25.0	-25.1	-28.1	-30.5	-30.6	-31.1	-31.2
31 Jan.D	1.7	-26.2	-26.2	n.d	n.d	n.d	n.d	n.d
32 Jan.N	1.5	-26.6	-26.4	n.d	n.d	n.d	n.d	n.d
1 Feb.D	1.94	-25.4	-26.3	n.d	n.d	n.d	n.d	n.d

[a] D: The samples were collected in daytime.

[b] N: The samples were collected in nighttime.

[c] n.d: The stable carbon isotope values are not quantified because the $\delta^{13}C$ values occurred at a concentration less than the limit of quantification.

Table S5. Pollutant concentration (μg m^{-3}) limits corresponding to the air pollution index

API[a]	Concentrations				
	SO_2(Ave.D)[b]	NO_2(Ave.D)	PM_{10}(Ave.D)	CO(Ave.H)[c]	O_3(Ave.H)
50	0.050	0.080	0.050	5	0.120
100	0.150	0.120	0.150	10	0.200
200	0.800	0.280	0.350	60	0.400
300	1.600	0.565	0.420	90	0.800
400	2.100	0.750	0.500	120	1.000
500	2.620	0.940	0.600	120	1.200

[a] The samples were classified into polluted days when API≥200, while the others were classified into non-polluted days.

[b] Daily average value

[c] Hour average value

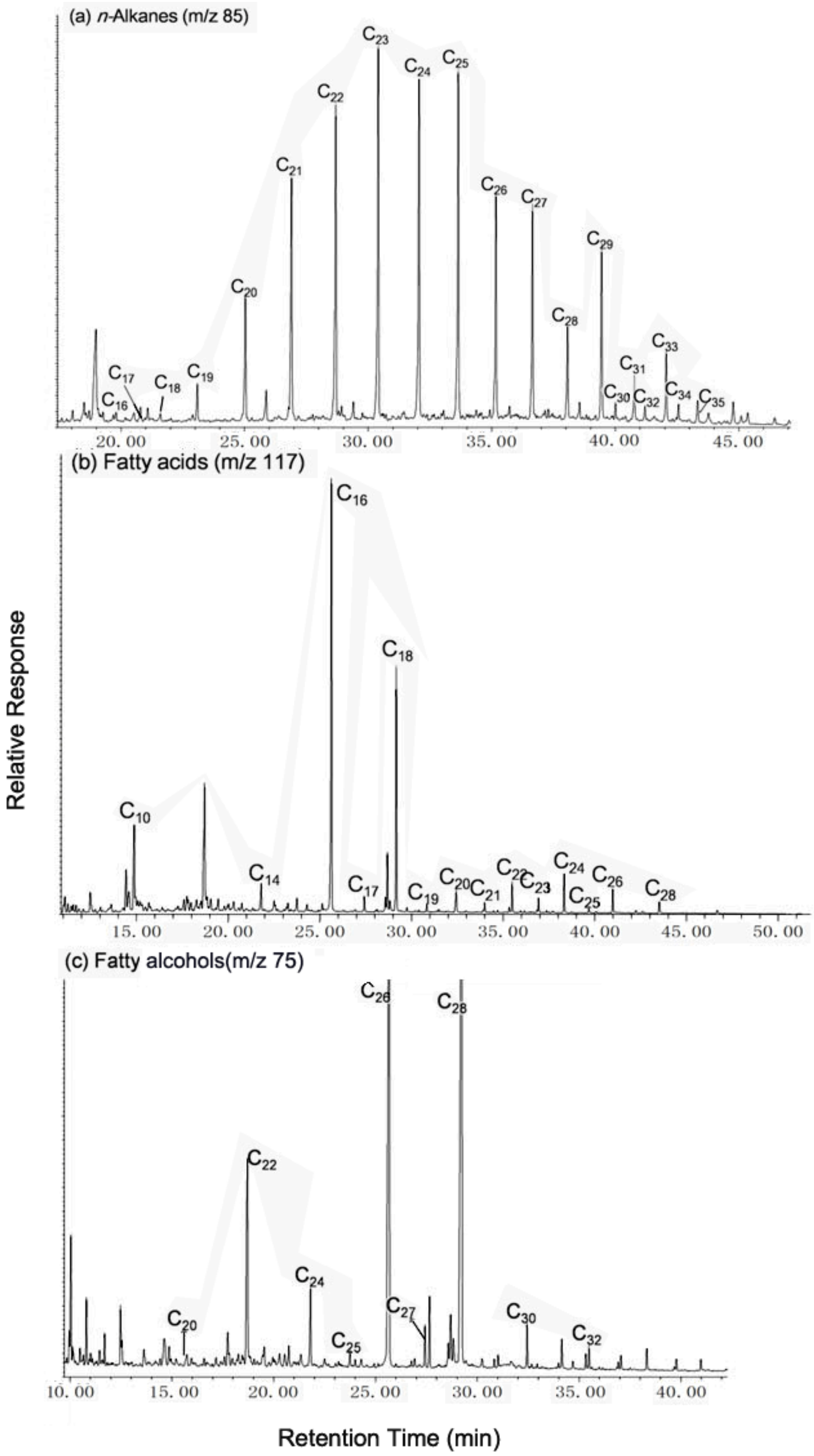

Figure S1. Salient features of the gas chromatography-mass spectrometry for aerosol samples from Beijing. (a) m/z 85 plots for *n*-alkanes, (b) m/z 117 plots for fatty acids, (c) m/z 75 plots for fatty alcohols.

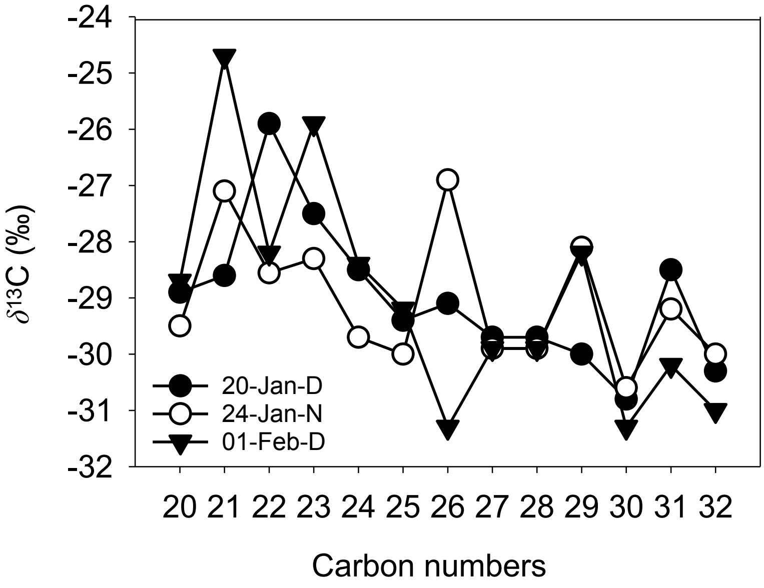

Figure S2. The δ^{13}C distributions of individual n-alkanes. D and N mean samples were collected in daytime and nighttime, respectively.

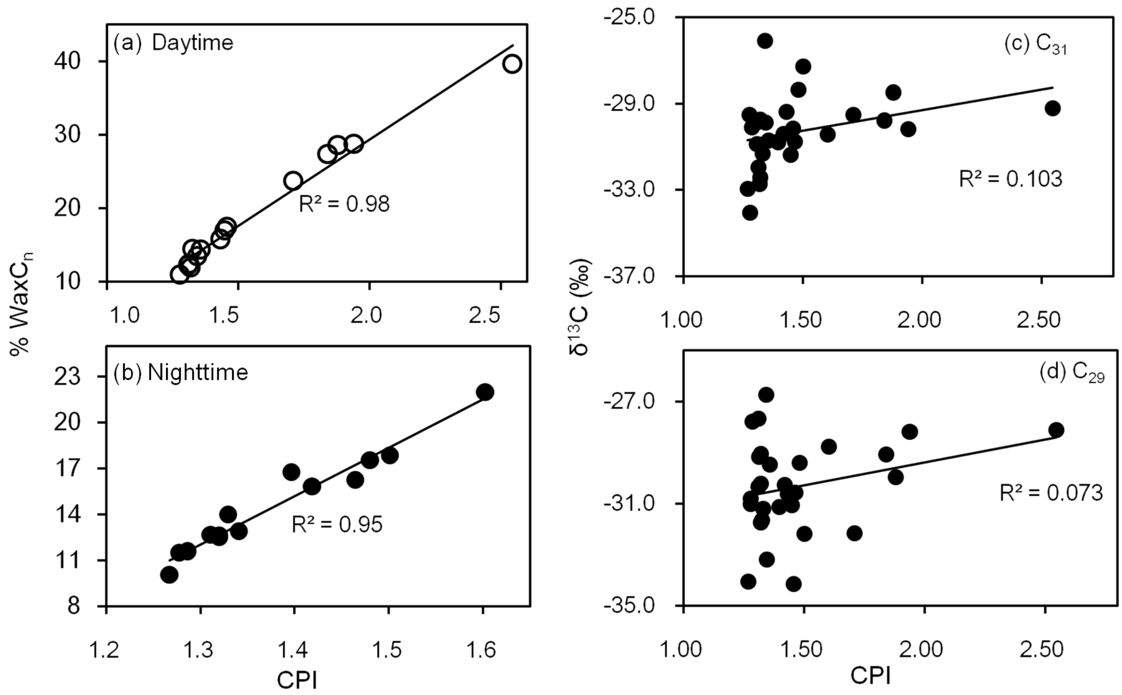

Figure S3. The correlation relationships between CPI and %WaxC_n, $\delta^{13}C$ in *n*-alkanes. (a) The correlation between CPI and %WaxC_n in daytime, (b) the correlation relationship between CPI and %WaxC_n in nighttime, (c) the correlation between CPI and C_{31}, and (d) the correlation between CPI and C_{29}.

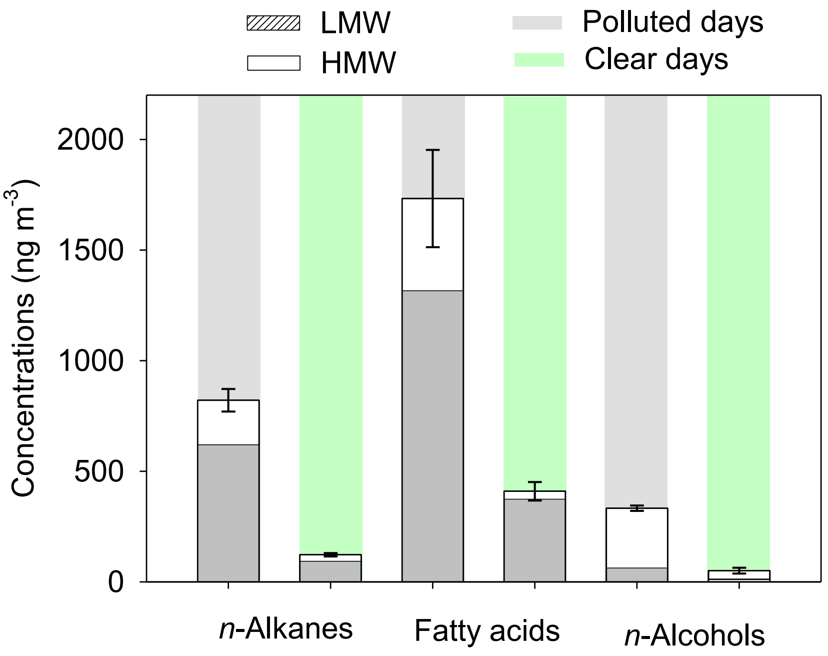

Figure S4. Mean concentrations of *n*-alkanes, fatty acids and *n*-alcohols on polluted and clean days.

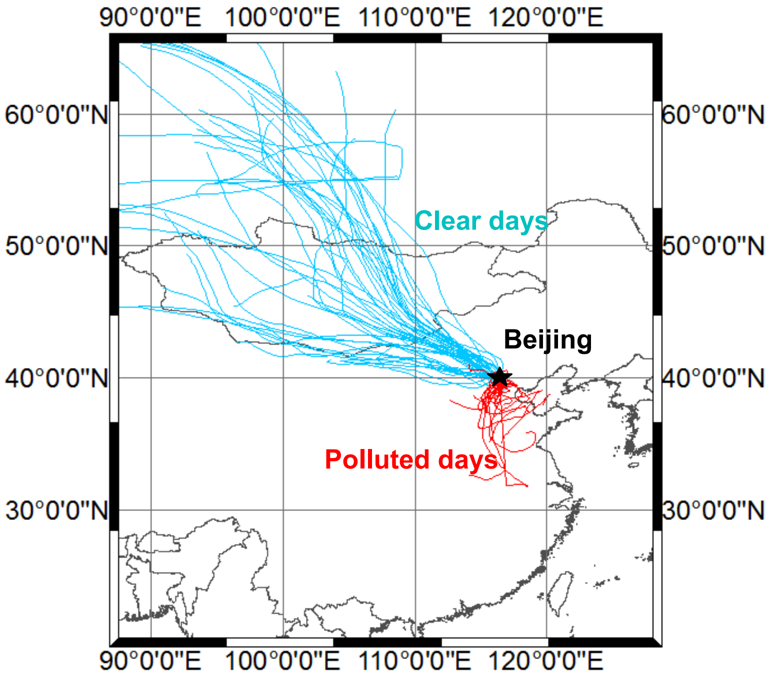

Figure S5. Three-day air mass back trajectories occurring at 200 m (a.s.l.) in Beijing during polluted days (blue lines) and clear days (black lines). Back trajectories at 200 m height were calculated every 3 h using NOAA HYSPLIT. The map was drawn by ArcGIS 9.3.
